# PathwayMatrix: visualizing binary relationships between proteins in biological pathways

**DOI:** 10.1186/1753-6561-9-S6-S3

**Published:** 2015-08-13

**Authors:** Tuan Nhon Dang, Paul Murray, Angus Graeme Forbes

**Affiliations:** 1 Department of Computer Science M/C 152, University of Illinois at Chicago, 851 S. Morgan, Room 1120, Chicago 60607-7053, IL, USA

**Keywords:** Pathway visualization, Biological networks, Matrix visualization, Binary Relationships

## Abstract

**Background:**

Molecular activation pathways are inherently complex, and understanding relations across many biochemical reactions and reaction types is difficult. Visualizing and analyzing a pathway is a challenge due to the network size and the diversity of relations between proteins and molecules.

**Results:**

In this paper, we introduce *PathwayMatrix*, a visualization tool that presents the binary relations between proteins in the pathway via the use of an interactive adjacency matrix. We provide filtering, lensing, clustering, and brushing and linking capabilities in order to present relevant details about proteins within a pathway.

**Conclusions:**

We evaluated *PathwayMatrix *by conducting a series of in-depth interviews with domain experts who provided positive feedback, leading us to believe that our visualization technique could be helpful for the larger community of researchers utilizing pathway visualizations. *PathwayMatrix *is freely available at https://github.com/CreativeCodingLab/PathwayMatrix.

## Background

Biological pathways consist of interactions and biochemical reactions between sets of proteins and other biomolecules resulting in a product or a change in cellular state or activity [1]. For instance, some pathways describe metabolic processes which break down carbohydrates, fats and proteins to create energy. Other pathways show how cells respond to external stimuli, such as immune cells detecting and responding to bacteria or viruses. Still other pathways produce changes in gene regulation, such as pathways that initiate mediated cell death. Each of these pathways contains a series of biochemical reactions between proteins and biomolecules, such as a phosphorylation reaction ("RAF1 phosphorylates MEK1") where one protein adds a phosphate group to another, changing the protein's state.

While some pathways are well characterized, researchers continually make discoveries that result in the identification of new interactions between pairs or proteins, or that explain the structure of, or refine existing knowledge related to, a molecular network under different cellular conditions. This growing volume of "biological network" data, available via public databases [2-5], is in part fueled by the development of high-throughput experimental techniques and the standardization of biological network data formats, allowing scientists to more rapidly generate and collect biological network data [6-8]. As the scale and complexity of this network data grows, along with the potential for error, there is a need for novel representations that address critical tasks in curating and analyzing these datasets [9].

The biggest challenge in presenting pathways is their complexity. Pathways may contain hundreds of proteins or small molecules, which form hierarchical and nested complexes that participate in a series of multistage reactions of various kinds. Representing complexity while also enabling researchers to see higher order patterns is a significant challenge [10].

Human generated pathway diagrams have many benefits, and many pathway visualization tools mimic the hand-made style of presenting pathway data. However, pathway complexity makes it difficult to view higher-order patterns. Other tools use an approach that instead attempts to reduce complexity by presenting an abstracted view of pathway data, where complex interactions are reduced to binary (and sometimes directed) relations between proteins. Given this abstraction, these representations do not show multistage reactions, protein complexes, or protein state transitions. All interactions are treated as binary relationships, without consideration of reactions that include multiple inputs and multiple outputs. This simplification aims to make it possible for researchers to see broader patterns within a pathway, without being distracted by details.

A set of rules (the simple interaction format, or SIF) were developed to reduce interactions to pairwise relationships [5]. Since SIF interactions are always binary it is not possible to fully represent all complex relationships, and this translation is lossy in general. The SIF format specifies nodes and interactions only, and SIF is convenient for building a graph from a list of interactions. It also makes it easy to combine different interaction sets into a larger network, or to add new interactions to an existing data set. The main disadvantage is that this format does not include any layout information. Nonetheless, SIF network data remains useful for certain types of bioinformatic applications that require pairwise interaction [11]. The SIF format can be easily imported into popular network analysis tools, such as *Cytoscape *[12], or *BioFabric *[13].

Our application, *PathwayMatrix*, represents binary relations between pairs of proteins and biomolecules in a network to enable the identification of modules or sub-networks within large pathways. In this paper, we describe *PathwayMatrix*, which includes: colored glyphs that indicate reaction types; a simplification technique in which dense parts of a pathway network are grouped and simplified; linked Venn diagrams and arc diagrams that complement the network data shown in the matrix; and interaction techniques that allow for data overlays and on-demand lensing to reveal details of complex pathways. This paper introduces these features and our motivations for incorporating them in our application, provides clear examples of why such a visualization is needed, and demonstrates some of the benefits this method has over traditional node-link diagrams for certain pathway visualization tasks.

## Related work

There exist a variety of approaches to represent biological pathway data. These include, among others: a) human generated layouts of curated pathways; b) variations on node-link diagrams that are presented with automated layouts; and c) overviews depicting binary relationships between proteins and molecules in pathways [14,15]. While these approaches enable a variety of analysis tasks, they also have significant limitations, including a lack of scalability to large networks and a tendency toward visual clutter that impedes critical pathway analysis tasks, such as identifying sub-networks, detecting the importance of a protein in a pathway, and understanding causality.

Abstracting pathway data to binary relationships can help to address the problem of scalability. Visualizations that focus on binary relationships can display visualizations of large, complex biological networks that may include thousands of proteins and complexes. Several visualization tools use this abstracted binary approach, including PCViz [5], VisANT [16], BioFabric [13], and Compressed Adjacency Matrices [17]. PCViz and VisANT both produce traditional node-link diagrams with force-directed layouts. VisANT is capable of showing very large networks, and includes interactive brushing and linked views that allow an analyst to explore subsets of nodes within a network. PCViz lets researchers search for interactions that include target proteins of interest, allowing for more focused inquiry into how a protein interacts with other proteins. However, since both of these approaches use conventional graph layout algorithms, the spatial positioning of nodes is not necessarily optimized for biological questions. As with many graph visualizations, as the number of nodes increase, the "hairball problem" inhibits analysis.

BioFabric [13] is a novel layout approach, which attempts to "comb the hairball" and provide layouts for easier understanding of complex graphs. It accomplishes this by adapting the node-link diagram to a matrix format, and by using lines to represent nodes, where intersections between horizontal and vertical lines represent links between nodes. However, using BioFabric to trace causal patterns across a sequence of reactions remains challenging.

The compressed adjacency matrix [17] was built specifically for gene regulatory networks, an essentially different class of biological network. Gene regulatory networks, in particular, contain few cycles, which are common in molecular activation networks. This representation extends adjacency matrices in a way that includes paths containing more than two nodes, which enables the visualization of causal relationships from one starting protein to another.

Broadly speaking, these approaches abstract details, but in the process some important information is lost. In some cases, misinterpretation of the representation is possible, such as when several proteins with shared binary interactions may appear to all interact at once, while in reality they interact in multiple, distinct stages. Finally, many critical tasks that could be addressed through abstraction are not targeted by these tools. These tasks include easily discerning the multiple complexes and reactions in which a given protein participates and detecting elements with similar functionality.

In the following sections, we introduce our tool, *PathwayMatrix*, providing scalable views of pathway data that reduce complexity while retaining important pathway information. All example biological pathways are found from the Reactome Pathway Database and are encoded using the BioPAX format.

## Methods

Visualizing and analyzing large pathway networks is a daunting task due to the network size and variety of types of connections between proteins in the pathway. To avoid the "hairball" problem, we introduce a novel matrix visualization that presents some advantages over node-linked diagrams for certain pathway visualization tasks.

### Overview of pathway visualization tasks

Over the course of many in-depth discussions with systems biologists, molecular biologists, and bioinformatics researchers, we identified a set of design goals to address some important visualization tasks relevant to biological pathway datasets.

**T1. Provide a comprehensive overview of the binary relations between proteins within a pathway: **We represent these binary relations in an adjacency matrix rather than via the more conventional node-link graph. This abstraction of the pathway data can help analysts identify modules and sub-networks within a pathway without the visual clutter that graph layouts can create, for example, due to edge crossings in large, dense networks.

**T2. Visualize multiple types of relations between proteins: **It is very common to find multiple relationships between a pair of proteins or biomolecules. Our glyph design maximizes visual impact by clearly highlighting these relationships, which is especially useful when exploring large networks.

**T3. Detect proteins with similar functionality in a pathway: **We define a measure of protein similarity in a particular pathway network based on the set of relationships between the proteins. Using this measure, we are able to order proteins by their similarities and create clusters of proteins having identical functionality.

**T4. Create interactive maps: **For biologists, it can be important to zoom in to see details about particular proteins or groups of proteins of interest. *PathwayMatrix *provides lensing, brushing and linking capabilities allowing users to perform this task.

### Binary interaction patterns in a BioPAX model

Based on the needs of bioinformatics researchers who we helped clarify the goals of the application, we look only at protein-protein relations, as other biological elements, such as small molecules are less important for pathway analysis tasks. The five protein-protein interaction types which include directionality that can be explored via *PathwayMatrix *are indicated by the following phrases, where *p*1 and *p*2 are the two proteins participating in a binary relation:

*p*1 controls-state-change-of *p*2

• *p*1 controls-phosphorylation-of *p*2

• *p*1 controls-transport-of *p*2

• *p*1 controls-expression-of *p*2

• *p*1 catalysis-precedes *p*2

Additionally, our application displays three protein-protein interaction types where directionality does not play a role:

• *p*1 in-complex-with *p*2

• *p*1 interacts-with *p*2

• *p*1 neighbor-of *p*2

The distribution of these relation types in pathways varies significantly in different pathways.

**Figure 1:**
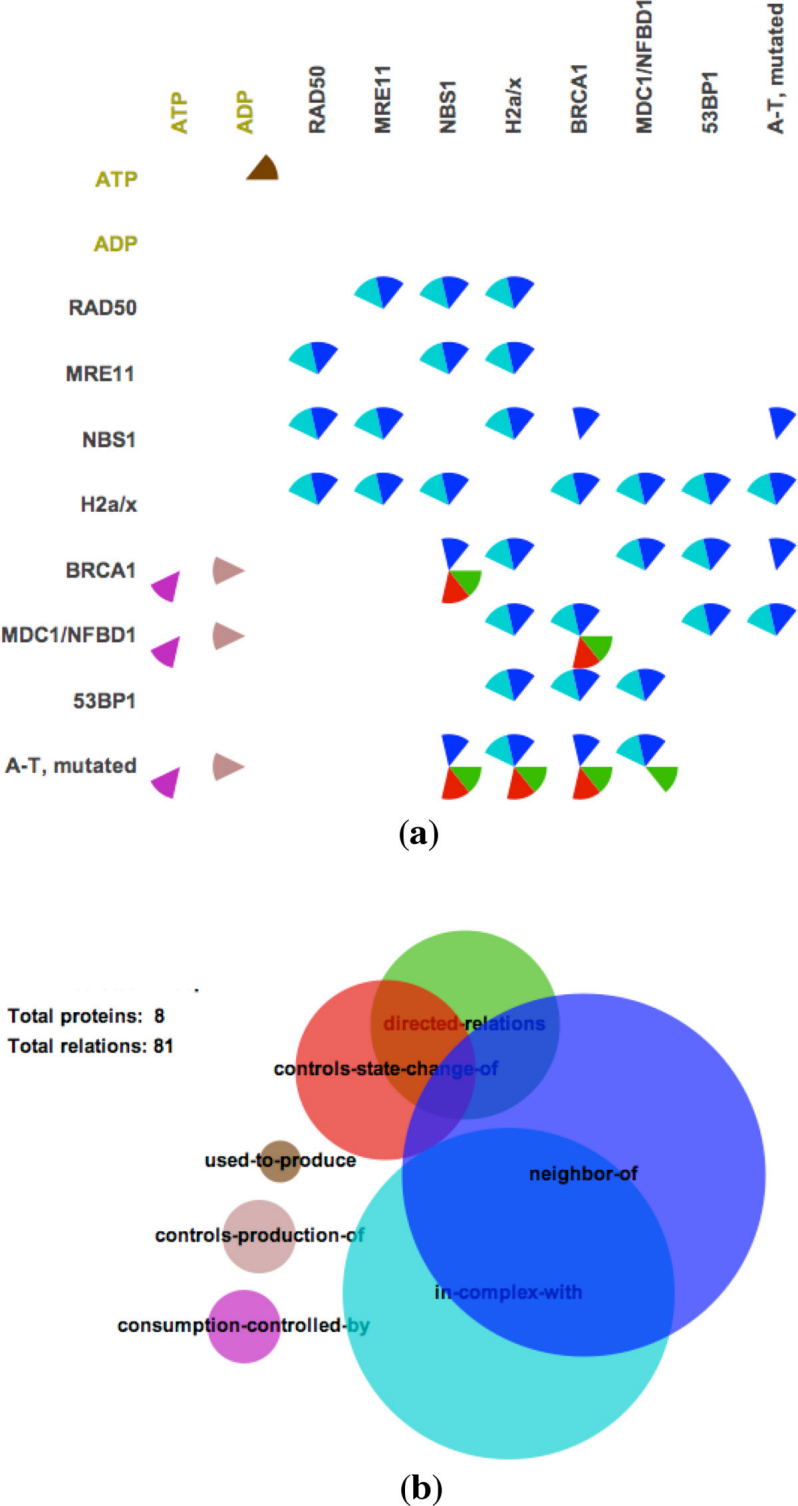
**Visualizing the *ATM Mediated Phosphorylation of Repair Proteins *pathway: (a) Matrix view of protein binary relations (b) Venn diagram summarizing protein binary relations**.

Our choice of the glyphs used to represent binary relationships (**T2**), depicted in Figure 1, follows the guidance on pop-out effects by Maguire et al. [18]. Pop-out effects enable faster visual searching for a target among unalike distractors. Based on extensive studies in psychophysics, the four most effective visual channels are color, size, shape, and orientation [19,20], where color has the strongest pop-out effect [18]. Therefore, in *PathwayMatrix *we use color as the primary visual channel to encode different types of binary relationships between proteins within a pathway. Orientation is used as a secondary visual channel, since, as discussed below, our interaction techniques alter the size of glyphs and because it would be difficult to use shapes (as will become apparent later in the paper). In some pathways not all of the binary interaction types are present.

Figure 1, shows an example of *PathwayMatrix *for the *TM Mediated Phosphorylation of Repair Proteins *pathway. In each cell of the matrix, we use circular sectors, divided similarly to a pie-chart, to indicate relations between two proteins, where each sector in circle is given a color to indicate interaction type. A modified Venn diagram is also supplied to provide an overview of the types of interactions within the pathway. The size of circles in this diagram represents the frequency of different relations in the pathway, and overlapping areas provide an overview of how often relations coexist in the pathway. We use the same color encoding for the Venn diagram and the matrix. For example, red represents "controls-state-change-of" relationships and blue represents "neighbor of" relationships.

The inclusion of interaction types through colored circular sectors and the linked diagram are both unique to this work. Together they better integrate important contextual information into the main view of the proteins, addressing a significant limitation in other abstracted pathway representations. The Venn diagram is constructed based on Wilkinson's algorithm [21], which provides an approximation of the computationally-hard problem of computing circle overlaps.

### Ordering proteins in a matrix

**Figure 2:**
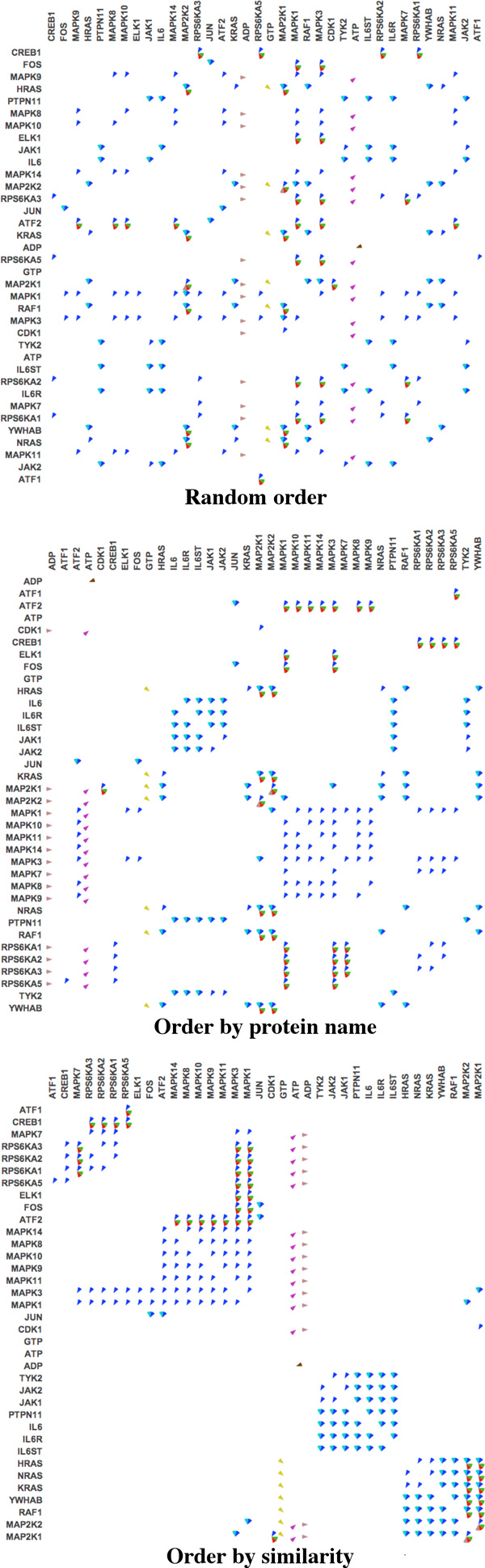
**Different protein orderings in the *RAF-MAP Kinase Cascade *pathway**.

Figure 2 shows different ways of ordering proteins in the *RAF-MAP Kinase Cascade*.

We implemented ordering algorithms in an effort to reveal higher-level patterns in large pathways, such as subnetworks and clusters of related proteins. Matrix reordering is a widely studied topic, and it has applications to many different fields such as sociology, psychology, and economics [22]. However, existing matrix ordering algorithms are not applicable to our application, since in our formulation each cell of the matrix contains a set of relations, rather than a single value. Therefore comparing two elements in the matrix involves comparing the set of common and uncommon relations. We developed a novel measure of similarity between two proteins (*T3*), and we use this metric to order the proteins in the matrix.

Our ordering algorithm starts with a random protein and adds successive proteins to the ordering based on their similarity. This ordering continues until all genes are ordered. The dissimilarity of two proteins is computed as follows:

Let <*P*_1_, *P*_2_, ..., *P_n _*> be the set of proteins in a pathway and *R_ij _*be the set of relations between *P_i _*and *P_j_*. The dissimilarity of two proteins *P_i _*and *P_j _*is then computed by the following equation:

(1) $$Dis(P_{i},\;P_{j})=\overset{n}{\underset{k=1}{\sum }}\left(|U_{ijk}|-w\;*\;|C_{ijk}\right)|)$$

where *C_ijk _*is the set of common relations of *R_ik _*and *R_jk _*with respect to protein *P_k_*, which is defined by *C_ijk _*= *R_ik _*∩ *R_jk_*.

*U_ijk _*is the set of uncommon relations of *R_ik _*and *R_jk _*with respect to protein *P_k_*, which is defined by *U_ijk _*= (*R_ik _*− *R_jk_*) *∪ *(*R_jk _− R_ik_*).

To compute *|U_ijk _|*, we use the equation:

(2) $$\left|U_{ijk}|=|R_{ik}|+|R_{jk}|\;-\;2\;*\;|C_{ijk}\right|$$

For example, if *R_ik _*= *{A, B, C, D} *and *R_jk _*= *{A, B, X}*. Then, *C_ijk _*= *{A, B} *and *U_ijk _*= *{C, D, X}*. Therefore, we have *|U_ijk _| *= *|{A, B, C, D}|*+*|{A, B, X}| − *2 *∗ |{A, B}| *= 4 + 3 *− *2 *∗ *2 = 3.

Replacing Eq. 2 into Eq. 1, we have:

(3) $$Dis\left(P_{i},\;P_{j}\right)=\overset{n}{\underset{k=1}{\sum }}\left(\left|R_{ik}|+|R_{jk}|\;-\;\left(w+2\right)\;*\;|C_{ijk}\right|\right)$$

Notice that *Dis*(*P_i_, P_j_*) can be a negative integer if there are no uncommon relations but many common relations between *P_i _*and *P_j _*with respect to the third protein *P_k_. w *is a parameter which allows the viewer to control the importance of common binary relations over uncommon binary relations. In other words,

• When *w *= 0, we compare the two proteins based on the set of uncommon relations (to other proteins).

• When *w *is a large integer, we consider the two proteins similar if they have many common relations (to any other proteins).

**Figure 3:**
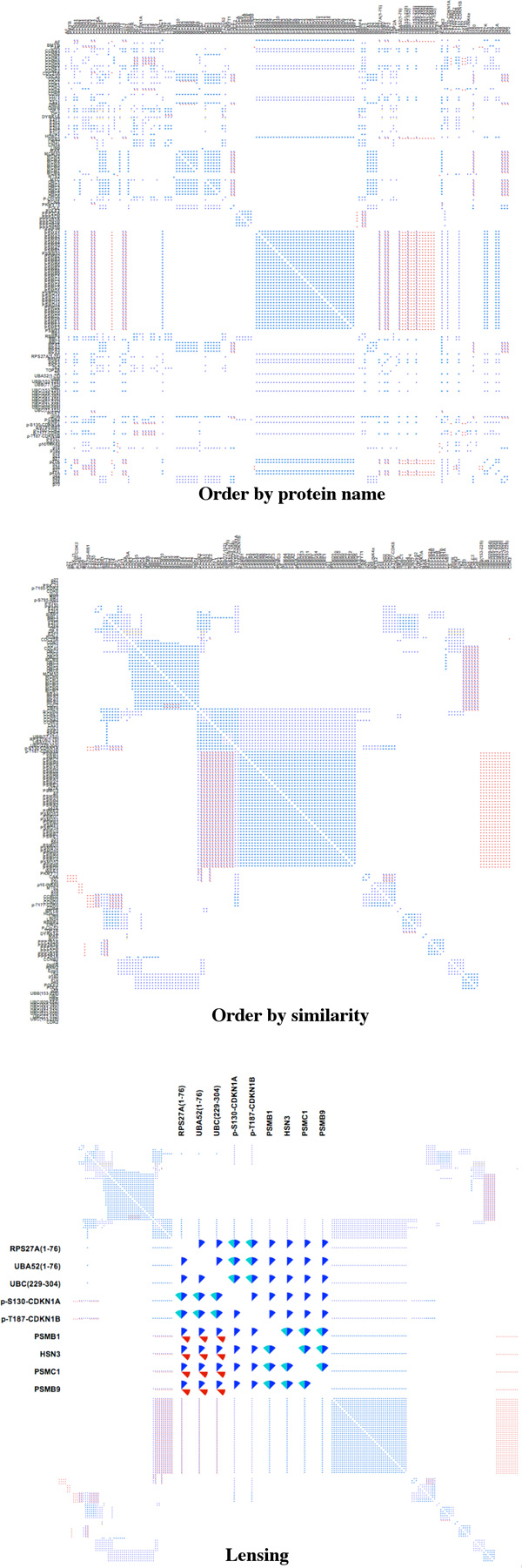
**Matrix view of different protein orderings and lensing (last panel) in the *Rb-E2F *pathway**.

The three panels of Figure 3 show the effects of ordering proteins in different ways. The top panel shows proteins ordered by their name. This helps to bring proteins in the same family together when their names are similar (i.e., only the ID numbers at the end of the protein name are different). However, this naming convention is not always accurate. Additionally, some proteins that belong to different protein families may perform the same functionality within a pathway. As illustrated in the middle panel, patterns become apparent when ordering proteins by similarity are applied.

### Lensing over the matrix

When the data includes hundreds of proteins, each circular sector becomes too small to decipher. We provide a lensing tool that helps the viewer to zoom in on a group of relationships in a specific area of the matrix without losing the overall context. The bottom panel of Figure 3 shows an example of lensing on the *Rb-E2F *pathway. This pathway contains 9,016 binary relations between 156 proteins and small molecules. Lensing helps address scalability limitations when viewing large networks.

Significantly, we use animation to show smooth transitions between different ordering schemes, which can improve perception by maintaining object constancy [23]. Animation is also helpful when applying lensing on the matrix. As it is difficult to describe animation in static images, we invite the reader to refer to the video in our associated supplementary materials.

### Grouping proteins together

**Figure 4:**
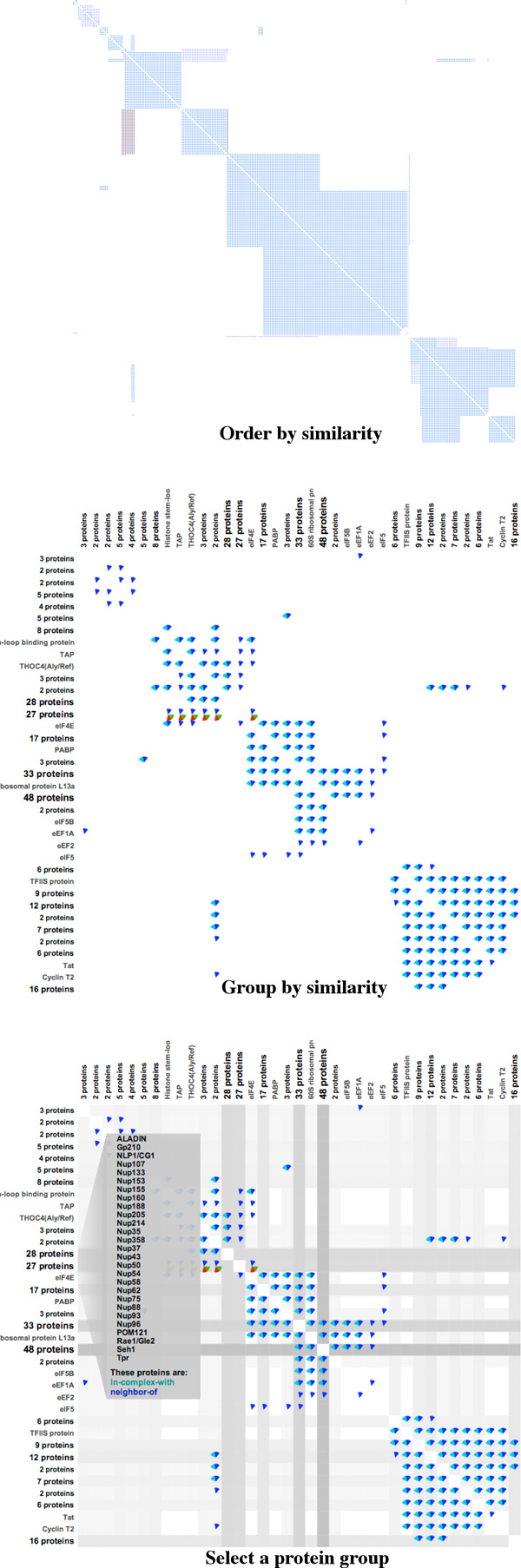
**Matrix view of ordering by protein similarity (top panel) and grouping (middle and last panel) in the *RNAP II Pre-transcription Events *pathway**.

Even with lensing, large networks can be difficult to view. For instance, in Figure 4 we show another example of the *RNAP II Pre-transcription Events *pathway. This pathway contains 31,866 relations between 294 proteins. The first panel shows an adjacency matrix with proteins ordered by similarity. To manage the scale of this network, we introduce a new technique called "group by similarity." The grouping algorithm is based on the idea of the leader algorithm, where assignments are made in a way that is similar to the iterative assignments in the k-means algorithm [24], except with only one pass through the data. Thus, the computational complexity of the leader algorithm is considerably less than that for k-means.

Our grouping algorithm proceeds as follows:

1 We initialize the leader list *L *= *∅*.

2 For each protein *P_i_*, we find the leader protein in L which has the same set of relations as *P_i _*has.

3 If we could not find a leader protein satisfying this condition, we make *P_i _*a new leader and add *P_i _*into *L*.

4 Otherwise, we add *P_i _*to the follower list of this leader protein.

5 Repeat steps 2 to 4 for all proteins *P_i_*.

When groups are computed, we then "collapse" nodes within a group into a single node, as shown in the middle panel of Figure 4. Each point in the matrix now represents relationships between proteins or groups of proteins, where the members of a group all share identical sets of relations. We indicate group size through a text label. Group size can also be indicated through the darkness of rows and columns in the matrix.

A viewer can explore proteins or relations in a protein group by simply clicking on its name, as depicted on the left side of the last panel in Figure 4 (where we have selected a group of 27 proteins). In this example, *PathwayMatrix *has automatically grouped all proteins in the *nucleoporin *families which are the constituent building blocks of the *nuclear pore complex (NPC) *[25].

Additionally, this cluster also contains other proteins outside the nucleoporin family, such as *ALADIN, NLP1, POMP121, Rae1, Seh1*, and *Tpr*. The 27 proteins were grouped together because they each have the same set of relation types as all the other proteins in this group.

### Displaying the hierarchy of protein complexes

Complexes consisting of many proteins are common in pathway datasets. In addition, these complexes are hierarchical, with several complexes nested together. However, abstracted views showing only binary relations lose this important hierarchical information. We introduce a new approach which shows complexes in a secondary linked view, and also allow hierarchy information to be overlaid on the adjacency matrix view.

**Figure 5:**
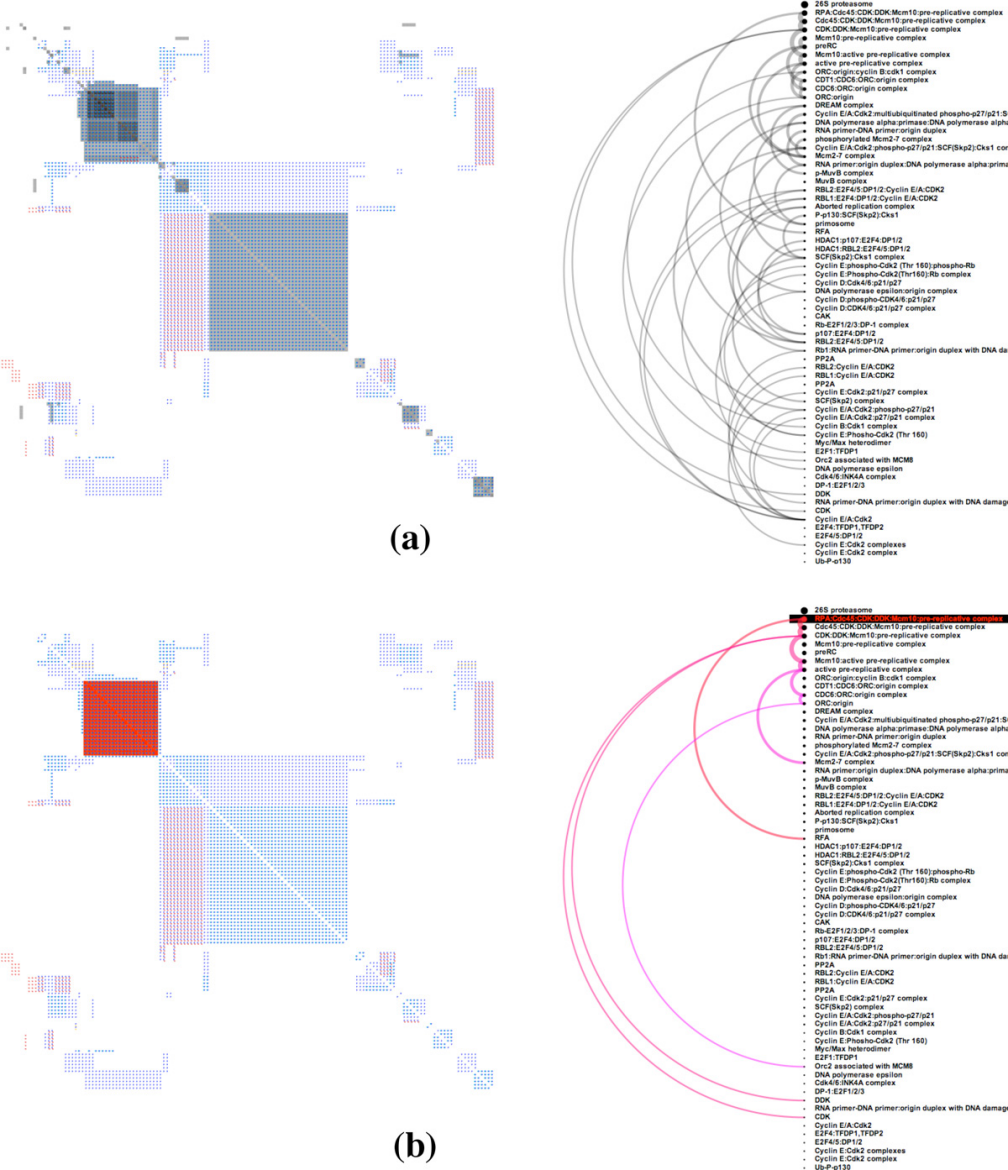
**Visualizing the hierarchy of protein complexes in the Rb-E2F pathway: (a) All descendant relationships between complexes (b) Descendant relationships between selected complexes**.

The linked view, shown in Figure 5, is placed next to the adjacency matrix, and uses a list to display the hierarchy of complexes in a pathway. Complexes in the list are ordered by size (the total number of proteins in complex). Given that complexes can be nested within other complexes, a nested complex will always appear below its parent in this list.

The list is paired with an interactive arc diagram. Arcs connect proteins and complexes that are in the same hierarchy. When a protein or complex is selected from the list, the arcs connecting members of its hierarchy are highlighted, and those complexes are also highlighted in the adjacency matrix. Arc thickness is determined by the size of the sub-complex, and the saturation of arcs indicates how many levels away the sub-complexes are from the selected complex. The arc diagram is used instead of, for example, a simple indented list because any one protein or complex may belong to many different parent complexes, making a typical hierarchical representation infeasible.

Viewers can overlay the complex structure in the pathway matrix as depicted in Figure 5(a). Darker areas highlight intersections of proteins and sub-complexes belonging to multiple complexes. When the user hovers the mouse over a complex, the arcs recursively highlight descendants (sub-complexes) of the selected complex as depicted in Figure 5(b).

### Implementation and requirements

*PathwayMatrix *is implemented in Java and built on top of Processing framework. It is cross-platform, with a minimum requirement of Java 7. *PathwayMatrix *uses Paxtools [26] for the loading and management of BioPAX data. The application, source code, and sample data are provided via our Github repository, located at https://github.com/CreativeCodingLab/PathwayMatrix.

## Results and discussion

### Comparison to other visualization tools

**Figure 6:**
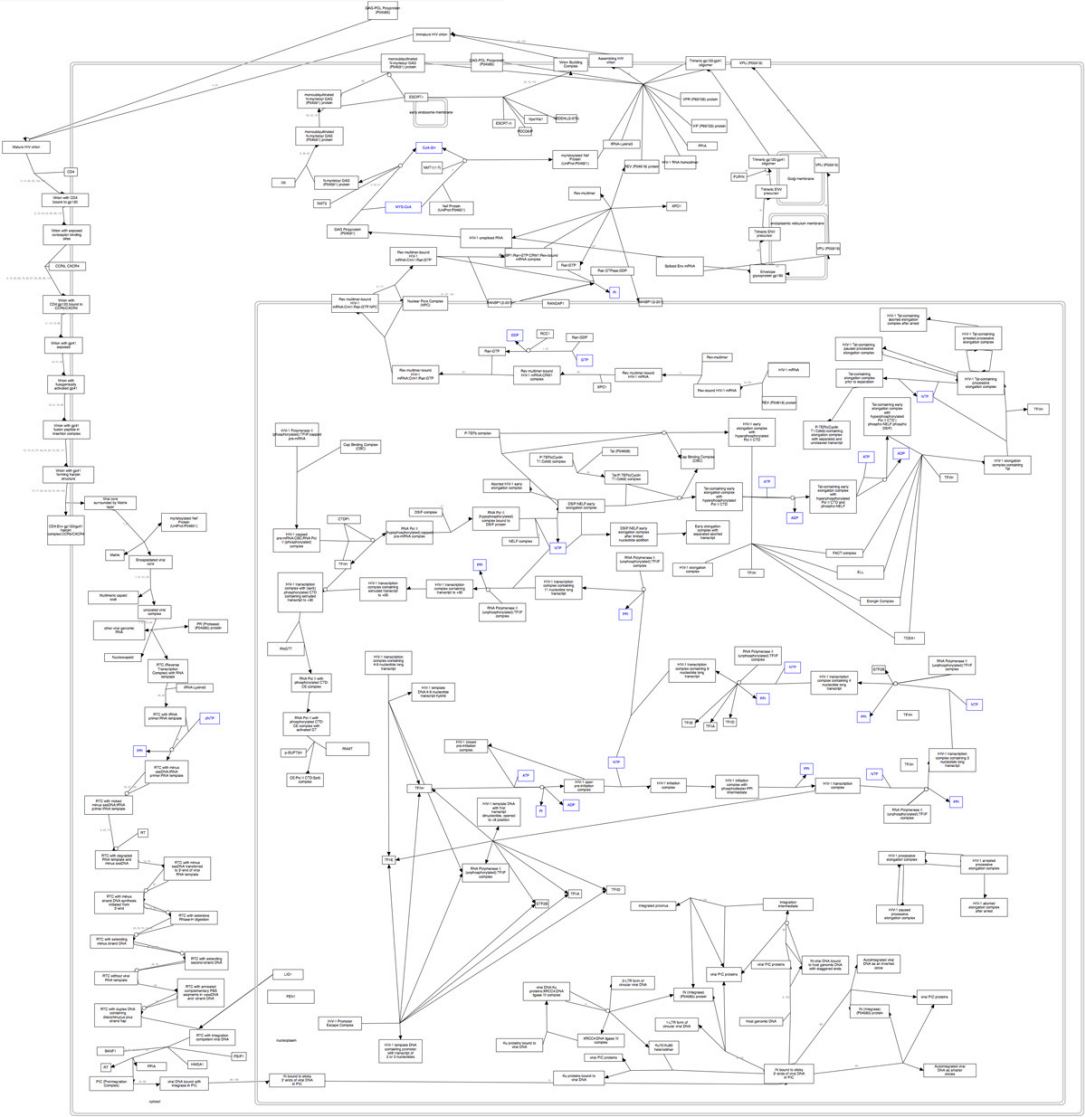
**Human generated diagram of the *HIV life cycle *by different tools downloaded from WikiPathways **[27].

In this section, we compare *PathwayMatrix *to other popular tools for viewing pathway data. We use the *HIV life cycle *to show differences between these tools; this pathway can be downloaded from WikiPathways [27] or Reactome [28]. Figure 6 shows the human generated diagram of this pathway. Though these hand-made figures are sometimes used in pathway visualizations, such as Entourage [29], they are not appropriate for every type of analysis task. (The pros and cons of these human generated pathway diagrams have been discussed above in the *Background *section.)

Effectively visualizing this data can be difficult since we may need to consider a wide array of *n*-ary relationships. However, representing this data as a simple binary network between proteins can also be very helpful because it allows the user to quickly see higher order patterns. Moreover, it becomes more straightforward to can apply conventional graph algorithms to visualize and analyze this binary networks. For instance, *Cytoscape *features layout algorithms that attempt to minimize edge crossings, and *BioFabric *uses a novel network presentation method that represents nodes as horizontal line segments, one per row.

Our technique is inspired by the compressed adjacency matrix technique that was introduced by Dinkla et al. [17] for representing relationships in gene regulatory networks. This technique is related to our work, since it also makes use of matrix representation, but is different in some essential ways. Pathway data includes many types of relationships, while compressed adjacency matrices are optimized for three types of relations (promotion, inhibition, or both) in a gene regulation network, with color being used to indicate these relationships. That is, it would be difficult to extend this technique to handle multiple types of binary relations between proteins; it would require too many colors to clearly encode the 8 different kinds of relationships and all of their combinations. Therefore, we do not include compressed adjacency matrices in our comparison.

**Figure 7:**
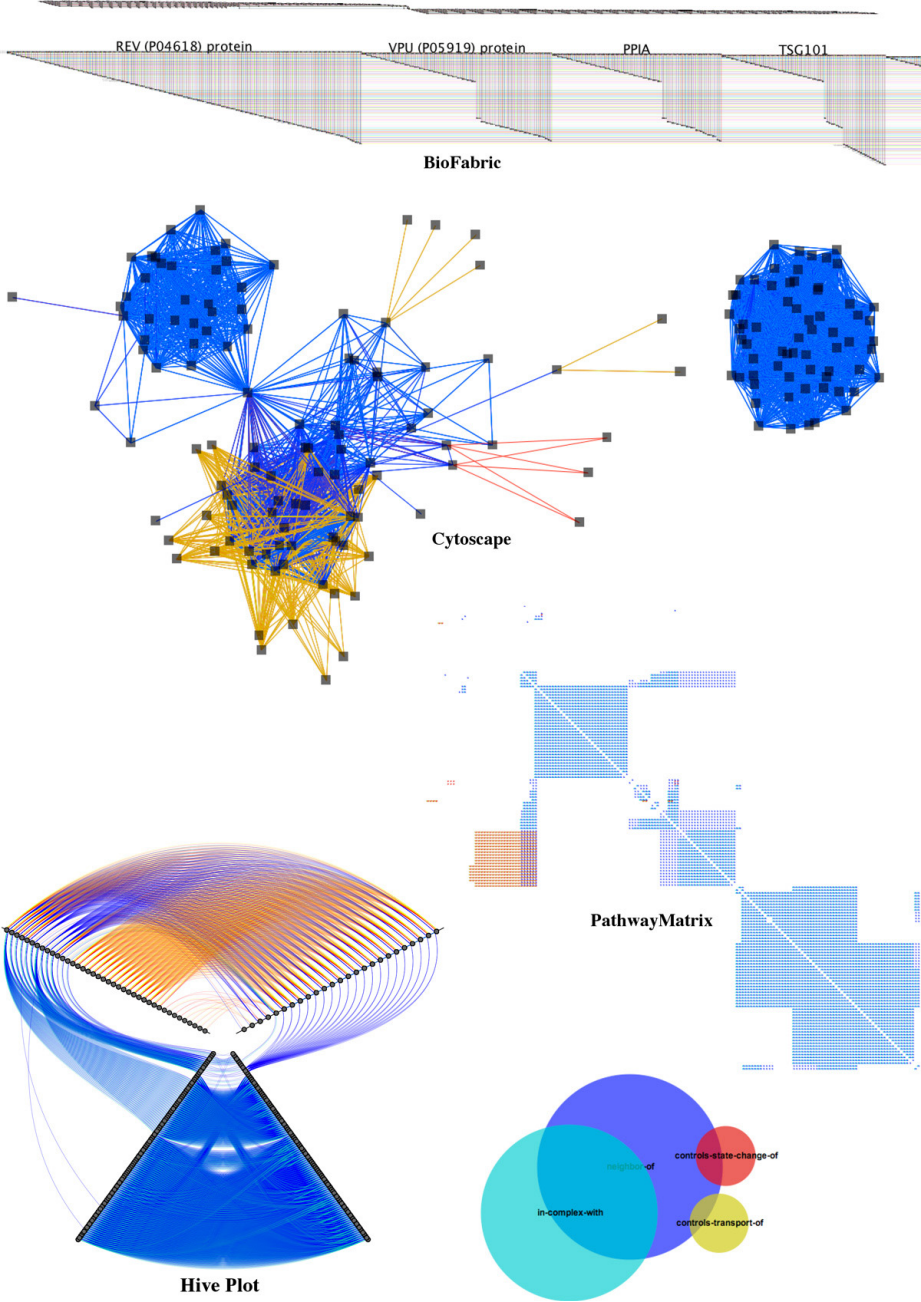
**Visualizing protein binary relationships of the *HIV life cycle *by different tools: BioFabric (top panel), Cytoscape (middle panel), Hive Plot (bottom left panel), and *PathwayMatrix *(bottom right panel)**.

Figure 7 shows an example of these tools on a medium-sized pathway network, the *HIV life cycle*, which contains 11,337 binary relations of 176 proteins. In the top panel, we show the *BioFabric *representation of this pathway, with an overview (that is difficult to reproduce in a figure) and a close-up of one small portion of the network. *BioFabric *may not be ideal for visualizing this pathway data. Because pathway networks are sparse and because multiple relations may exist between a pair of proteins, *BioFabric *visualizations become very long and cannot fit onto the screen to provide a comprehensive overview of the data. In addition, the *HIV life cycle *network contains two disconnected components which are difficult to discern from the overview of *BioFabric *shown at the top of the top panel.

In the middle panel of Figure 7, we show *Cytoscape*'s force directed layout for the same data. In this view, the two components of the network are clearly separated: the left network is associated to the early stage of the *HIV life cycle *while the right network is associated to the late stage of the *HIV life cycle*.

The bottom left panel of Figure 7 shows a *Hive Plot *[30] of the data. Each dot represents a protein in the pathway, and each link represents a binary relation from one class to another. Links are colored according to their relation type. Hive Plot defines a linear layout for nodes, arranging them along radial axes based on their connectivity degree (higher degree nodes are positioned toward the outer edge of the radial layout). Proteins are separated into three groups: nodes with more outgoing links (top right axis containing 25 proteins), nodes with more incoming links (top left axis containing 40 proteins), and nodes with the same number of incoming and outgoing links. The axis for the last group (at the bottom) is duplicated to reveal relationships within this group. In this view, the two components of the network are not clearly separated. However, we can quickly notice that the relations "controls-state-change-of" (in red) and "controls-transport-of" (in yellow) only exist between proteins in the first two groups (on the top left axis and the top right axis).

The two disconnected components of the *HIV life cycle *are easily discerned in the *PathwayMatrix *shown at the bottom right of Figure 7: the top left part of the matrix is associated to the early stage of the *HIV life cycle *while the lower right part of the matrix is associated to the late stage of the *HIV life cycle*. At the bottom, we also show the Venn diagram of the four most prevalent binary relationships between proteins in the pathway :"neighbor-of" is in blue; "incomplex-with" is in cyan; "controls-state-change-of" is in red; and "controls-transport-of" is in yellow. This same color encoding is consistently used in other tools for comparison.

**Figure 8:**
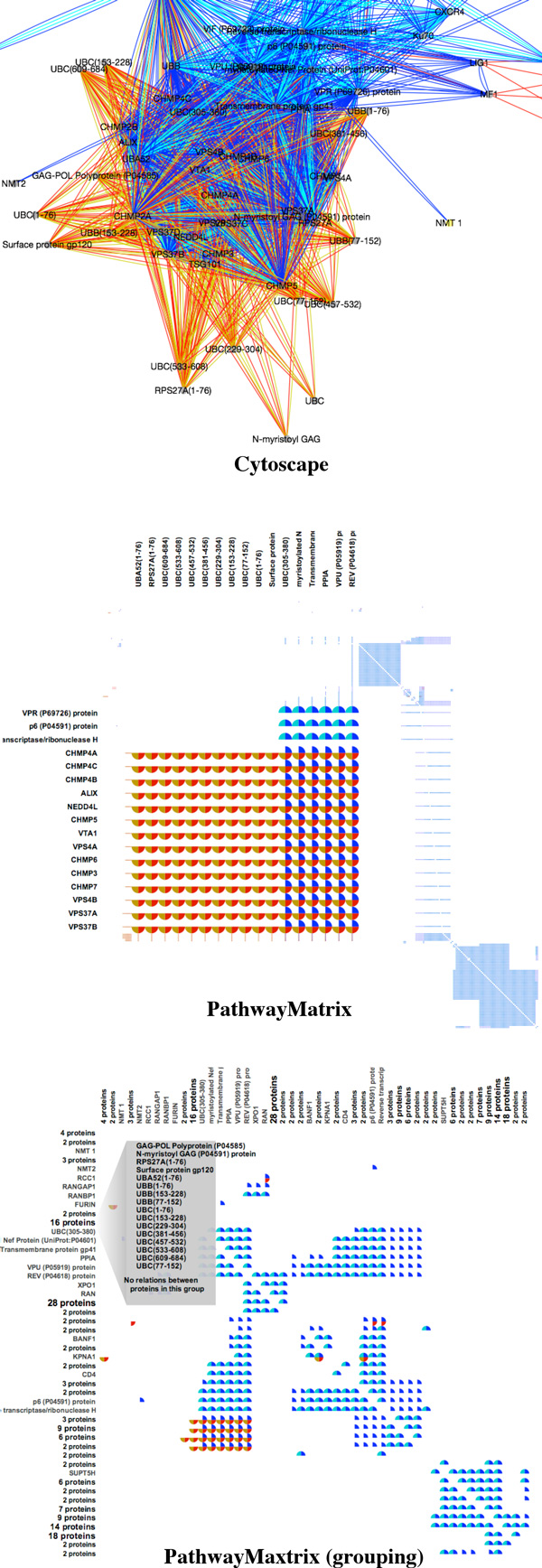
**Zooming in on a sub-network of the *HIV life cycle*: Cytoscape (top panel) and *PathwayMatrix *(middle panel)**. Grouping similar proteins in *PathwayMatrix *(last panel).

The top panel of Figure 8 shows a zoomed-in view of the *HIV life cycle *network in *Cytoscape*. Here we see visual clutter due to edge crossings in a dense part of the network. In the middle panel of Figure 8, the same region of the network is shown using the lensing tool in *PathwayMatrix*. The relationships between proteins are much easier to interpret in *PathwayMatrix*. In the last panel, we show that we can further reduce the complexity of the matrix view by collapsing similar rows and columns together. The visualization now displays the binary relationships between different groups of proteins. When we mouse over a group name, *PathwayMatrix *displays all proteins in that group, as shown in the last panel of 8. The 16 proteins of the selected group (containing proteins in the *U BA, U BB*, and *U BC *families) have no relationships with any other proteins in this group, but they have the same set of relations to other proteins or protein groups in the pathway.

It is difficult to make a comparison between *PathwayMatrix *and techniques that utilize node-link diagrams as a matrix representation is optimal for different tasks than a node-link diagram. Nonetheless, this comparison shows that our tool can provide important structural information that is lost when using other techniques. Our tool is not meant to replace existing network views, but instead to provide an alternative visualization technique for biological networks that may enhance particular analysis tasks.

### Expert feedback

We presented our *PathwayMatrix *to three domain experts (one a molecular biologist, one a systems biologist, and the third a bioinformatics researcher). Each of the experts are very familiar with the various tools used to visualize biological pathways. We conducted interviews regarding the potential relevance of the *PathwayMatrix *to their research tasks, and we further asked them for comparisons of to alternative visual representations.

All three experts were intrigued by our tool and responded positively overall. Both biologists noted that the protein-level view provided a nice overview of the high-level features in protein-to-protein relationships and indicated that the options for sorting and clustering would be useful for identifying relevant regions of the pathway. One of the experts also indicated that the tool could be useful as a "debugging" tool to detect files that may contain incomplete or inaccurate information about a biological pathway.

One expert used *PathwayMatrix *to inspect a pathway taken from the *Reactome *Pathway Database. Using the grouping features in the protein-level view, it quickly became apparent that the data file actually encoded two unconnected pathways, something that was obscured in the more cluttered views often associated with node-link representations. He noticed that our tool also highlighted certain protein identifiers that could not be resolved, making it easier to curate pathway data files.

Both the biologists and the bioinformatician also pointed out limitations of matrix representations. Since we decompose *n*-ary relationships (for example, biochemical reactions which involve multiple input/output proteins and biomolecules) into binary relations, the chronological ordering is lost. Although it is easier to visualize causality in node-link visualizations, such as ChiBE [31], tracing causal patterns across a sequence of biochemical reactions remains challenging, especially for larger pathways, in both matrix and node-link representations. Visualizing causality is an interesting future direction of this work.

In future work, we would like to be able to handle a much larger amount of data and to include data from multiple pathways. Although grouping proteins by their functionality in the network significantly reduces its complexity, the reduced (grouped) matrix is still rather complex when given large amounts of data. In this case, we could further cluster the reduced matrix if we relax the grouping criteria to allow for differences between grouped proteins. Grouping could occur at many levels, and a linked-view dendrogram of protein names could help explain how proteins are clustered into groups at different levels.

## Conclusions

*PathwayMatrix *presents an alternative visual representation of biological pathways using an interactive matrix representation instead of a node-linked diagram. A pathway contains multiple interaction types between proteins. The glyphs inside each circular cell can indicate concurrent relations between a pair of proteins. We maximize the pop-up effects by using colors and orientations of circular sectors. This is helpful for larger networks when the space assigned for each cell in the matrix is limited.

*PathwayMatrix *supports interactive capabilities to help users interested in exploring very dense pathway networks. The ability to order by protein arranges similar proteins so that they appear together in the visualization. Smooth lensing allows the viewer to focus on a particular protein or a group of proteins that is of interest. Grouping similar proteins provide a more compressed view of the entire network. Finally, users can overlay information about protein complexes on the top of the matrix to visualize their nested structure.

Although this paper focuses largely on alternative representations to a node-link diagram, we believe that it may be useful to present our visualization techniques alongside (instead of in replacement of) a node-link diagram. Although one advantage of our representations is that they are less cluttered than node-link representations, we plan to further investigate the scalability of our system and to explore other ways of interactively compressing or expanding parts of the pathway as needed, something that may prove important for very large pathways containing more than a few hundred proteins or for loading multiple pathways simultaneously, a feature we plan to implement in the future.

## Competing interests

The authors declare that they have no competing interests.

## Authors' contributions

TND, PM, and AGF conceived of the interactive visualization technique and its application to biological pathways. PM provided the overview of pathway visualization tasks. TND implemented all aspects of the prototype visualization tool, including the definitions of the similarity metrics and the lensing and grouping methods; he also provided the comparison to existing tools. TND, PM, and AGF drafted, read, and approved the final manuscript.

## Declarations

This publication is supported by the DARPA *Big Mechanism *Program under ARO contract WF911NF-14-1-0395.

This article has been published as part of *BMC Proceedings *Volume 9 Supplement 6, 2015: Proceedings of the 5th Symposium on Biological Data Visualization: Part 2. The full contents of the supplement are available online at http://www.biomedcentral.com/bmcproc/supplements/9/S6.
